# A longitudinal assessment of appetite loss and nutritional care among postoperative patients in Vietnam

**DOI:** 10.3389/fnut.2023.1008636

**Published:** 2023-03-23

**Authors:** Tu Huu Nguyen, Nguyet Thi Ta, Anh Kim Dang, Tham Thi Nguyen, Vu Anh Trong Dam, Carl A. Latkin, Cyrus S. H. Ho, Roger C. M. Ho

**Affiliations:** ^1^Faculty of Nursing and Midwifery, Hanoi Medical University, Hanoi, Vietnam; ^2^Institute for Preventive Medicine and Public Health, Hanoi Medical University, Hanoi, Vietnam; ^3^Institute for Global Health Innovations, Duy Tan University, Danang, Vietnam; ^4^Faculty of Medicine, Duy Tan University, Danang, Vietnam; ^5^Bloomberg School of Public Health, Johns Hopkins University, Baltimore, MD, United States; ^6^Department of Psychological Medicine, Yong Loo Lin School of Medicine, National University of Singapore, Singapore, Singapore; ^7^Institute for Health Innovation and Technology (iHealthtech), National University of Singapore, Singapore, Singapore

**Keywords:** postoperative appetite, appetite score, anorexia, postoperative patients, Vietnam

## Abstract

**Background:**

Post-operative appetite loss is an important complication affecting surgical outcomes. It has been estimated that nearly 60% of patients having gastrointestinal or major elective surgeries suffer from malnutrition. Appetite refers to the physical desire for food appetite, and losing appetite after surgery may result in a decrease in body weight, impairment of intestinal absorption and eventually, malnutrition among postoperative patients. This study aims to assess appetite status and other relevant factors among abdominal postoperative patients in Vietnam.

**Methods:**

A cross-sectional study was conducted on 169 abdominal postoperative patients from June 1st to August 30th, 2016 at Hanoi Medical University Hospital, Hanoi, Vietnam. Appetite score was computed by using the Council on Nutrition Appetite Questionnaire (CNAQ). This study used GEE to account for the potential correlation of outcomes of the longitudinal assessment, assuming an independent correlation structure.

**Results:**

The primary and secondary outcome measures: highest average appetite score was recorded in the preoperative day and the score declined throughout seven-day duration. Patients who were female, under general anesthetics and being under open surgery tended to get lower appetite scores. The majority of patients had moderate to good appetite in both the preoperative day and seven days post-operation.

**Conclusion:**

Women should receive more care and help in regaining their appetite after surgery. Treatment for appetite loss through non-pharmaceutical measures should be prioritized. Interventions that increase the appetite of patients after abdominal surgery should be targeted on patients being under general anesthetic as well as open surgery and be undertaken with caution.

## Introduction

Nutrition for surgical patients is a major health concern worldwide. It has been estimated that nearly 60% of patients having gastrointestinal or major elective surgeries suffer from malnutrition (1–3). This problem is attributable to many reasons such as the decrease in oral food intake, impaired absorption caused by intestinal obstruction, postoperative fasting or gastric atony (4, 5). Previous evidence shows that suboptimal nutritional status puts postoperative patients at a higher risk of poor wound healing, decreasing the response of the immune system as well as delaying recovery (6, 7). In Vietnam, this condition can be met at up to 50% of patients after surgery (8, 9), which is higher compared to other developing countries (10).

The benefits of early enteral feeding for patients after abdominal surgeries have been previously underlined to reduce the consequences of malnutrition (11, 12). Patients ‘appetite has been regarded as a crucial indicator to examine the time for enteral feeding (13). Appetite refers to the physical desire for food appetite, and losing appetite after surgery may result in the decrease of body weight, impairment of intestinal absorption and eventually, malnutrition among postoperative patients (5, 14). This study used The Council on Nutrition Appetite Questionnaire (CNAQ) to measure comprehensive assessment of appetite based on eight perspectives including their appetite, feeling hungry or full, food taste, feeling nausea, number of meals per day, and mood among postoperative patients in Vietnam (15). The CNAQ is a psychometric scale that is easy to use, generic, and can be applied to a variety of clinical situations (16).

Appetite is associated with a number of factors, including demographic characteristics, psychobiological changes, and environments (17–19). However, it also depends on patients’ desire and preference, which varies across cultures and backgrounds. Understanding appetite loss and associated factors are therefore important to develop an appropriate feeding plan for abdominal postoperative patients. To date, little literature on appetite loss and its determinants among patients after abdominal surgery are available. Thus, this study aimed to assess appetite conditions and factors associated with appetite loss among patients after abdominal surgery. To the best of our knowledge, there is limited previous study comprehensively examining appetite loss and nutritional care in Vietnam. The improvement in the patient’s diet after surgery has remained inconclusive. Hence, this study aims to assess appetite loss and nutritional care as well as identify associated factor of anorexia among postoperative patients in Vietnam.

## Materials and methods

### Study design

A longitudinal study was conducted from June 1st to August 30th, 2016. Patients were recruited from two departments, Department of Surgery, and Department of Oncology and Palliative care, of Hanoi Medical University Hospital by convenience sampling. The eligibility criteria for choosing participants were: (1) being aged ≥18 years old; (2) undergoing intra-abdominal surgery; (3) having feeding in the duration of the first postoperative week; (4) agreeing to participate in the study, and (5) being able to provide verbal communication. The benefits and drawbacks of the study were clearly presented to the patients in the recruitment stage. Patients agreeing to participate in the study were informed study information and asked to give verbal informed consent. The confidentiality of patients was ensured, and their decision did not affect the treatment process. There were 169 patients participating in the study in total.

### Measurements and instruments

Patients were invited to participate in 20-min face-to-face interviews using structured questionnaires. Data collectors were well-trained researchers and physicians. The questionnaire included the following information: Socio-economic characteristics (gender, age, and occupation), Surgical – related characteristics and Appetite status assessment.

Surgical – related characteristics were collected from the medical record including participant’s weight and height, anesthetic method, American Society of Anesthesiologists (ASA) Physical status classifications, surgical method, and surgical position. ASA Physical Status Classification System was based on 6 levels, including ASA 1 (A normal healthy patient); ASA 2 (A patient with mild systemic disease); ASA 3 (A patient with severe systemic disease); ASA 4 (A patient with severe systemic disease that is a constant threat to life); ASA 5 (A moribund patient who is not expected to survive without the operation); ASA 6 (A declared brain-dead patient whose organs are being removed for donor purposes) (20). In addition, surgical history and the 1st postoperative (PO) flatulence were self-reported by patients.

Appetite was assessed by the Council on Nutrition Appetite Questionnaire (CNAQ) (15). The instrument assesses eight perspectives including their appetite, feeling hungry or full, food taste, feeling nausea, the number of meals per day and their mood. Each perspective was rated based on a five-point scale. The total score ranged from 8 to 40, in which the total score lower or equal 16 points indicated anorexia, from 17 to 28 was moderate appetite, >28 was a good appetite. Patients’ appetite was assessed before surgery and each day after surgery.

### Statistical analysis

Data was analyzed by STATA 15.0 (Stata Corp. LP, College Station, TX, United States). The information on appetite status was presented using descriptive statistics. We used frequency, percentage to describe the number of patients in each group of appetite status (anorexia, moderate appetite, good appetite) and mean, standard deviation (SD) to measure scores of appetites. In order to identify the differences in socioeconomic characteristics, surgical – related characteristics among anorexia and non-anorexia patients, we used the Chi-square test (for qualitative variables) and t-test (for quantitative variables). Multivariate generalized estimating equations (GEE) regression was utilized to identify factors associated with the CNAQ score. We used GEE to account for the potential correlation of outcomes of the longitudinal assessment, assuming an independent correlation structure. To control covariates, independent variables in the multivariable regression model included socioeconomic characteristics (age, gender, occupation), and surgical – related characteristics (time having the disease, surgical history, anesthetics method, surgical method, surgical position, first time of flatulence PO). We also estimated robust standard errors, adjusting for the clustering within the longitudinal assessment. value of *p* less than 0.05 was used to consider statistically significant.

### Ethics approval and consent to participate

The protocol of this study was approved by the Institutional Review Board of Hanoi Medical University (4,820/QĐ-ĐHYHN). Informed consent was also obtained from the participants.

## Results

### Socioeconomic characteristics

Table 1 presented the individual characteristics of the participants. The study was conducted among 169 patients, in which the percentage of male patients was 44.3%. There were 73.4% of patients aged less than 60 years old, and the mean age was 51.5. The figure for farmers, workers, and businessmen was the highest (62.7%).

**Table 1:** Individual characteristics of participants.

Characteristics		*n*	%
Age	<60	124	73.4
	≥60	45	26.6
Gender	Male	75	44.4
	Female	94	55.6
Occupation	Farmer/Worker/Business	106	62.7
	Office workers	50	29.6
	Retire	13	7.7
BMI (kg/m2)	≤18.5	22	13.0
	18.5–24.9	127	75.2
	25–29.9	20	11.8
Duration of having the disease	<1 year	132	78.1
	≥1 year	37	21.9
Surgical history	No	91	53.9
	Yes	78	46.2
ASA	I	97	57.4
	II	70	41.4
	III	2	1.2
Anesthetics method	General anesthesia	141	83.4
	Local anesthesia	28	16.6
Surgical method	Laparoscopy	122	72.2
	Open surgery and others	47	28.8
Surgical position	GI system	69	40.8
	Others	100	59.2
First time of flatulence PO	≤24 h	128	75.7
	>24 h	41	24.3

### The general information about the disease

Most of the patients were in normal nutritional status, comprising 75.2%. In terms of anesthetic methods, the highest number was general anesthesia (83.4%). The percentage of patients treated by laparoscopy method was highest (72.2%), while open surgery and others accounted for 28.8%. The proportion of patients having a surgical position in the gastrointestinal (GI) system (40.8%) was smaller than that in others (59.2%). Almost all the patients had flatulence on the first day after surgery (75.7%).

Table 2 presents the appetite status of the patient from the preoperative day to the postoperative days. Most of the patients had a good appetite before surgery, accounting for 65.5%. The percentage of patients having a good appetite reduced from the day before surgery recovered on the fifth day and continuously increased to the day before discharge. The percentage of patients having anorexia was the highest on the fourth day and the fifth day after surgery. In the sixth- and seventh-day post-operation, none of the patients suffered from anorexia.

**Table 2:** Appetite status of patient from the preoperative day to postoperative days.

Time	Appetite					
	Anorexia		Moderate appetite		Good appetite	
	*n*	%	*n*	%	*n*	%
Preoperative (*n* = 148)	2	1.4	49	33.1	97	65.5
First day PO (*n* = 169)	1	0.6	94	55.6	74	43.8
Second day PO (*n* = 169)	1	0.6	89	52.7	79	46.8
Third day PO (*n* = 89)	0	0.0	50	56.2	39	43.8
Fourth day PO (*n* = 57)	1	1.8	29	50.9	27	47.4
Fifth day PO (*n* = 33)	1	3.0	15	45.5	17	51.5
Sixth day PO (*n* = 19)	0	0.0	10	52.6	9	47.4
Seventh-day PO (*n* = 10)	0	0.0	5	50.0	5	50.0

**Figure 1:**
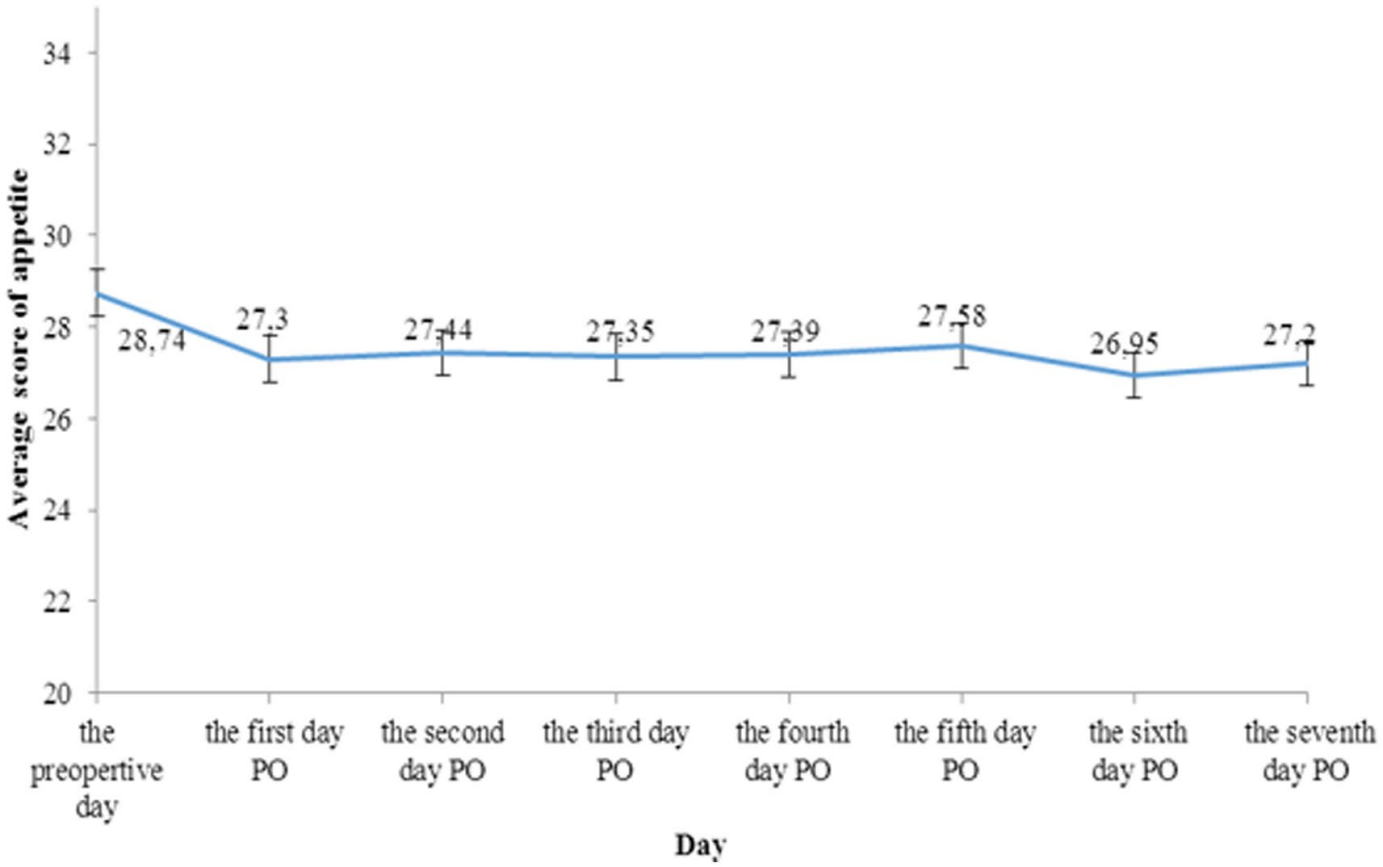
Appetite changes in preoperative day and postoperative days.

In Figure 1, there was a significant decline of average appetite score from the preoperative day to the first day PO (28.74 ± 3.67 vs. 27.3 ± 3.79, *p* < 0.05). The mean appetite score of patients was improved in the day before discharge when compared to the first day PO (27.88 ± 4.39 vs. 27.3 ± 3.79, *p* < 0.05).

Table 3 shows the appetite status of patients according to socioeconomic characteristics. A small percentage of patients had anorexia (0.6%) on the first postoperative day and in the day before discharge (0.6%), which only appeared in a group of people aged 60 and over. Most of the males had a good appetite on both days, accounting for 54.7% on the first day and 61.3% the day before discharge. The average appetite score in males was considerably higher than in females on the first day PO (28.2 ± 3.2 vs. 26.6 ± 4.1, *p* < 0.05) and on the day before discharge (28.8 ± 2.9 vs. 27.1 ± 4.0, *p* < 0.05). The majority of patients had normal status with BMI from 18.5 to 24.9 kg/m2 and this group had the highest prevalence of good appetite on the first day PO, comprising 46.5%. In terms of surgical history, the average score of appetite increased slightly from the first day of PO to the day before discharge in patients having a surgical history. Patients under local anesthesia had a higher mean appetite score than those having general anesthesia on both of first day PO and the day before discharge. The mean appetite score among patients having flatulence in the first 24 h PO was dramatically higher than patients having flatulence after the first 24 h PO in the first day PO (27.8 ± 3.4 vs. 25.6 ± 4.4, *p* < 0.05).

**Table 3:** Appetite status according to socioeconomic - and surgical - related characteristics.

Characteristics	Appetite in the first-day post-operation								Appetite in the day before discharge							
	AN		MA		GA		Score		AN		MA		GA		Score	
	*n*	%	*n*	%	*n*	%	Mean	SD	*n*	%	*n*	%	*n*	%	Mean	SD
Total	1	0.6	94	55.6	74	43.8	27.3	3.8	1	0.6	78	46.2	89	53.2	27.9	3.6
Age																
<60	0	0.0	65	52.4	59	47.6	27.5	3.7	0	0.0	56	45.2	68	54.8	27.9	3.6
≥60	1	2.2	29	64.4	15	33.3	26.8	3.9	1	2.2	22	48.9	22	48.9	27.7	3.9
Gender																
Male	0	0.0	34	45.3	41	54.7	28.2^a^	3.2	0	0.0	29	38.7	46	61.3	28.8^a^	2.9
Female	1	1.1	60	63.8	33	35.1	26.6	4.1	1	1.1	49	52.1	44	46.8	27.1	4.0
Occupation																
Farmer/Worker/Business	1	0.9	57	53.8	48	45.3	27.2	4	1	0.9	50	47.2	55	51.9	27.7	3.9
Office workers	0	0.0	26	52.0	24	48.0	27.7	3.4	0	0.0	20	40.0	30	60.0	28.4	3.0
Retire	0	0.0	11	84.6	2	15.4	26.2	3.9	0	0.0	8	61.5	5	38.5	27.4	4.0
BMI (kg/m^2^)																
≤18.5	0	0.0	14	63.6	8	36.4	26.3	4.4	0	0.0	10	45.5	12	54.5	28.4	3.7
18.5–24.9	1	0.8	67	52.8	59	46.5	27.5	3.8	1	0.8	58	45.7	68	53.5	27.8	3.7
25–29.9	0	0.0	13	65.0	7	35.0	27.4	3.3	0	0.0	10	50.0	10	50.0	27.7	3.2
Duration of having the disease																
<1 year	1	0.8	71	53.8	60	45.5	27.4	3.7	1	0.8	59	44.7	72	54.5	27.9	3.6
≥1 year	0	0.0	23	62.2	14	37.8	26.8	4.1	0	0.0	19	51.4	18	48.6	27.6	3.9
Surgical history																
No	1	1.1	46	50.5	44	48.4	27.7	3.8	0	0.0	45	57.7	33	42.3	27.2	3.6
Yes	0	0.0	48	61.5	30	38.5	26.8	3.8	1	1.1	33	36.3	57	62.6	28.5	3.6
ASA																
I	1	1.0	55	56.7	41	42.3	27	3.9	1	1.0	40	41.2	56	57.7	27.9	3.7
II	0	0.0	38	54.3	32	45.7	27.6	3.6	0	0.0	36	51.4	34	48.6	27.9	3.5
III	0	0.0	1	50.0	1	50.0	28.5	4.9	0	0.0	2	100.0	0	0.0	23.5	2.1
Anesthetics method																
General anesthesia	1	0.7	80	56.7	60	42.6	27.1	3.9	1	0.7	66	46.8	74	52.5	27.8	3.8
Local anesthesia	0	0.0	14	50.0	14	50.0	28.5	2.8	0	0.0	12	42.9	16	57.1	28.3	2.8
Surgical method																
Laparoscopy	1	0.8	65	53.3	56	45.9	27.6	3.6	1	0.8	52	42.6	69	56.6	28.2	3.5
Open surgery	0	0.0	29	61.7	18	38.3	26.5	4.1	0	0.0	26	55.3	21	44.7	27.1	4.0
Surgical position																
GI system	1	1.4	39	56.5	29	42.0	26.8	4.1	1	1.4	31	44.9	37	53.6	27.4	4.1
Others	0	0.0	55	55.0	45	45.0	27.6	3.5	0	0.0	47	47.0	53	53.0	28.2	3.2
First time of flatulence PO																
≤24 h	0	0.0	66	51.6	62	48.4	27.8^b^	3.4	0	0.0	56	43.8	72	56.2	28.1	3.3
>24 h	0	0.0	23	71.9	9	28.1	25.6	4.4	0	0.0	17	53.1	15	46.9	27.3	4.2

^a^Indicates a significant difference in average appetite score between Male and Female within the gender group in the first-day post-operation, *p* < 0.05. ^b^Indicates a significant difference in average appetite score between ≤24 h and >24 h within the group of first time have flatulence post-operation in the first-day post-operation, *p* < 0.05. AN, anorexia; ASA, American society of anesthesiologists; BMI, body mass index; GA, good appetite; GI, gastrointestinal; MA, moderate appetite; SD, standard deviation; PO, postoperative.

Table 4 shows the factors associated with appetite score among patients. Patients who were female (Coef = −0.32; 95%CI = −1.29; −0.05) and who had a general anesthetics method (Coef = −2.11; 95%CI = −4.04; −0.18) were less likely to have higher CNAQ scores. By contrast, patients who had other surgical methods were associated with having a higher appetite score compared to those having open surgery (Coef = 5.84; 95%CI = 2.37;9.33).

**Table 4:** Factors associated with anorexia among patients.

Characteristics	The council on nutrition appetite questionnaire score		
	Coef	95%CI	Value of *p*
Gender (Female vs. Male -Ref)	−0.32	−1.29; −0.05	0.01
Age (≥60 vs. <60 -Ref)	0.22	−2.34; 2.79	0.87
Occupations (Farmer/Worker/Business -Ref)			
Office workers	−0.04	−2.12; 2.04	0.97
Retire	0.47	−2.47; 3.41	0.76
Time having disease (≥1-year vs. <1 year -Ref)	−0.77	−3.33; 1.78	1.78
Surgical history (Yes vs. No -Ref)	−0.54	−2.07; 1.00	0.50
ASA (1 -Ref)			
2	−0.25	−2.20; 2.15	0.98
3	6.91	3.36; 10.45	<0.01
Anesthetics method (General vs. Local -Ref)	−2.11	−4.04; −0.18	0.03
Surgical method (Others vs. Open surgery -Ref)	5.84	2.37; 9.33	<0.01
Surgical position (Others vs. GI system -Ref)	1.78	−0.16; 3.72	0.07
First time of flatulence PO (>24 h vs. ≤24 h -Ref)	−0.31	−2.88; 2.26	0.81

ASA, American society of anesthesiologists; CI, confidence interval; Coef, coefficient; GI, gastrointestinal system; PO, postoperative; ref, reference group.

## Discussion

Our study revealed valuable findings on the appetite status of patients after having abdominal surgery. By using the Council on Nutrition Appetite Questionnaire, we found that the majority of participants had moderate to good appetite during the preoperative day to seven days after the operation. The highest average appetite score was recorded in the preoperative day and the score declined throughout the seven-day duration. This pattern could be explained by postoperative fatigue, which is the primary result of the stress response and inflammation (21, 22). Previous studies suggest that this symptom may lead to a secondary loss of appetite (23, 24). Patients usually start to rehabilitate and regain their appetite after receiving operation five days, and longer hospitalization periods may inhibit the return of the patients’ appetite (25, 26). In the context of Vietnam, the problem is more complicated because of the lack of available enteral products and experience of how to feed post-operative patients (9). Moreover, physician nutritionists do not recommend how to feed the individual patient and it is decided by the patient’s general practitioner (9).

Postoperative female patients tended to have a higher risk of anorexia compared to male patients. The mean appetite score of male patients was higher than females in both preoperative day and postoperative days, and this result is consistent with previous studies (27, 28). Gender differences in appetite-related areas have been previously presented and can be explained by the effect of hormones. Female sex hormones which include mainly estrogens can impact central and peripheral signals from several hormones controlling the feedback of eating, as well as mediate the estrogenic inhibition of eating during having meals (29). Higher neuronal activation among females may produce a greater inhibitory response to food cues than men and the differences in brain activation consequently create gender-specific appetite responses (30). Since our study did not take into account the mechanisms behind these gender differences, further studies should be conducted to identify the influence of gender on appetite response among postoperative patients.

Compared to those who had local anesthetics methods, patients under general anesthetics were less likely to have a higher appetite score. This can be explained by the fact that nausea, vomiting and dry mouth are the most common side effects following surgery under general anesthesia, which may reduce the appetite among patients (31). In addition, patients who were under open surgery had a lower score of CNAQ. Open surgeries with large open wounds may increase blood loss, pain, discomfort and recovery period for patients, which is associated with lengthy bed rest and inactivity (32, 33). This may increase the probability of loss of appetite, especially among those who underwent major surgeries (14, 34).

Generally, there are limited pharmaceutical treatments for postoperative appetite loss, among the most common and effective of which are Ghrelin and Rikkunshito (19). However, regular use of these medications may be problematic due to a lack of high-level evidence. For instance, while ghrelin has been extensively studied in recent time, most studies were randomized trials in phase II and therefore not suitable for routine use. Moreover, the scarcity of quantitative data synthesis for ghrelin was attributed to the variety of measurement endpoints, time since the operation, and protocols (35). Rikkunshito is a herbal medicine, whose positive effect has been proven on ghrelin levels. Although long-term use of rikkunshito is feasible, evidence from randomized control trials is not sufficient for public utilization (36, 37). Therefore, treatment for appetite loss through non-pharmaceutical measures should be prioritized. Besides conventional daily activities such as gum chewing and walking, nutritional interventions are increasing in popularity as they are convenient and often yield favorable results. Recommended nutrition plans included liquid supplements through spread out small meals or oral nutritional supplements (38, 39). Post-discharge nutritional counseling should also be provided for follow-up patients. Although no evidence has been acquired for patients who have undergone abdominal surgery, nutritional counseling has been found to improve appetite loss 1 month after discharge for esophagectomy patients, which is also open surgery (40). Therefore, it is highly recommended that a similar program be developed for abdominal patients all operative patients in general. Several implications can be drawn from this study. To begin with, since appetite status tends to decline in postoperative days, patients should undertake more activities that improve their intestinal health post-surgery, such as walking, chewing bubble gum, and having small meals (17, 41). This study also suggests that women should receive more care and help in regaining their appetite after surgery. Finally and most importantly, interventions that increase the appetite of patients after abdominal surgery should be targeted on patients being under general anesthetic as well as open surgery and be undertaken with caution.

### Strengths and limitations

The first strength of this study is that this is the first study assessing appetite loss and nutritional care, hence, this study provided a comprehensive picture of nutritional status among postoperative patients in Vietnam Secondly, this study used face-to-face interviews which allows more intensive data collection and understanding. Lastly, this study used validated international instruments (The Council on Nutrition Appetite Questionnaire) to assess the appetite of patients after surgery. However, there are several limitations should be considered in this study. The convenience sampling technique may limit the ability to generalize findings. In addition, causal relations cannot be established because of the cross-sectional design and information self-reported by patients may be inaccurate due to recall bias. Moreover, preoperative nutrition risk was not screening or assessment of nutritional status for the study participants, it might affect postoperative appetite.

### Conclusion

Among the patients after having abdominal surgery, appetite status may decrease from the preoperative day to the postoperative days. Patients who were female, under open surgery, having general anesthetics methods, and being under open surgery, tended to have lower appetite scores. This study suggests that women should receive more care and help in regaining their appetite after surgery. Treatment for appetite loss through non-pharmaceutical measures should be prioritized. Finally and most importantly, interventions that increase the appetite of patients after abdominal surgery should be targeted on patients being under general anesthetic as well as open surgery and be undertaken with caution.

## Data availability statement

The original contributions presented in the study are included in the article/supplementary material, further inquiries can be directed to the corresponding author.

## Ethics statement

The studies involving human participants were reviewed and approved by the Institutional Review Board of Hanoi Medical University. The patients/participants provided their written informed consent to participate in this study.

## Author contributions

THN and NT: survey conception. AD, TTN, and VD: data collection. THN, TTN, and VD: project management. TTN and VD: data interpretation. THN: drafting of the manuscript. CL, CH, and RH: reviewing and editing the manuscript. All authors contributed to the article and approved the submitted version.

## Funding

Research is supported by the Hanoi Medical University, NUS Department of Psychological Medicine (R-177-000-100-001/R-177-000-003-001/R177000702733) and NUS iHeathtech Other Operating Expenses (R-722-000-004-731).

## Conflict of interest

The authors declare that the research was conducted in the absence of any commercial or financial relationships that could be construed as a potential conflict of interest.

## Publisher’s note

All claims expressed in this article are solely those of the authors and do not necessarily represent those of their affiliated organizations, or those of the publisher, the editors and the reviewers. Any product that may be evaluated in this article, or claim that may be made by its manufacturer, is not guaranteed or endorsed by the publisher.

## Abbreviations

ASA, American Society of Anesthesiologists
CNAQ, The Council on Nutrition Appetite Questionnaire
GEE, multivariate generalized estimating equations
GI, gastrointestinal system
PO, postoperative
